# The Regulation of Inflammation by Innate and Adaptive Lymphocytes

**DOI:** 10.1155/2018/1467538

**Published:** 2018-06-11

**Authors:** David Alex Cronkite, Tara M. Strutt

**Affiliations:** ^1^College of Medicine, University of Central Florida, 6900 Lake Nona Boulevard, Orlando, FL 32827, USA; ^2^Immunity and Pathogenesis Division, Burnett School of Biomedical Sciences, College of Medicine, University of Central Florida, 6900 Lake Nona Boulevard, Orlando, FL 32827, USA

## Abstract

Inflammation plays an essential role in the control of pathogens and in shaping the ensuing adaptive immune responses. Traditionally, innate immunity has been described as a rapid response triggered through generic and nonspecific means that by definition lacks the ability to remember. Recently, it has become clear that some innate immune cells are epigenetically reprogrammed or “imprinted” by past experiences. These “trained” innate immune cells display altered inflammatory responses upon subsequent pathogen encounter. Remembrance of past pathogen encounters has classically been attributed to cohorts of antigen-specific memory T and B cells following the resolution of infection. During recall responses, memory T and B cells quickly respond by proliferating, producing effector cytokines, and performing various effector functions. An often-overlooked effector function of memory CD4 and CD8 T cells is the promotion of an inflammatory milieu at the initial site of infection that mirrors the primary encounter. This memory-conditioned inflammatory response, in conjunction with other secondary effector T cell functions, results in better control and more rapid resolution of both infection and the associated tissue pathology. Recent advancements in our understanding of inflammatory triggers, imprinting of the innate immune responses, and the role of T cell memory in regulating inflammation are discussed.

## 1. Introduction

Advances on several research fronts have significantly broadened our understanding of the triggers and modulators of inflammation. Of importance to this review, we now appreciate that at sites of infection, adaptive immune memory cells regulate innate inflammatory responses that contribute to the control of pathogens. Herein, potential means to modulate inflammation for the optimal generation of protective immunity through vaccination are discussed.

The ultimate goal of vaccination is to stimulate the generation of long-lived protective immunity without causing adverse clinical symptoms. Traditional vaccination strategies employing inactivated or attenuated pathogens or pathogen-derived protein antigens primarily target the generation of neutralizing antibody responses from B cells that act to prevent infection upon pathogen reencounter [1]. These regimes have been remarkably effective at mitigating the morbidity and mortality of a number of infectious diseases in vaccinated populations and most notably have led to the complete eradication of smallpox [2]. However, intracellular pathogens like influenza viruses (IAV) [3], human immunodeficiency virus (HIV) [4], and *Mycobacterium tuberculosis* [5, 6] have yet to be effectively controlled by neutralizing antibody-based vaccine approaches. Such pathogens either rapidly mutate external proteins that are targets for antibody or are not likely seen by antibody and are more effectively controlled by cell-mediated immune responses. The generation of protective T cell-mediated immunity through vaccination is appealing for pathogens like IAV that undergo antigenic shifts to evade neutralizing antibody given that T cells can recognize antigenic targets that are more conserved between strains. T cell-based vaccines against IAV may thus have the benefit of mediating universal protection against unforeseen and emergent pandemic strains of the virus [7], and they may potentially also eliminate the need for annual IAV vaccine reformulation. Inflammatory enhancing adjuvants have the potential to boost the efficacy of novel neutralizing antibody-based and T cell-based vaccines [8–11]. In order for such adjuvanated T cell-based vaccines to be efficacious and safe, they will need to target the induction of both pathogen-specific inflammation and adaptive immunity at relevant sites of infection.

## 2. There: The Regulation of Innate Inflammatory Responses by Pathogen

When a pathogen breaches the initial barriers of the skin or a mucosal surface, both soluble and cellular innate defense mechanisms are encountered and an inflammatory response is rapidly initiated. Some of the most potent soluble antimicrobial factors encountered include complement, lysozymes, defensins, mucins, lectins, cathelicidins, and lipocalins [12–15]. Several of these soluble antimicrobial mediators, such as activated complement components and lipocalin-2, are pluripotent, and in addition to performing antimicrobial functions, they amplify the inflammatory response triggered in resident sentinel immune cells upon pathogen sensing [13, 16, 17]. Within minutes to hours of detection of alarm signals, a “heightened alert” inflammatory transcriptional program ensues in sentinel innate immune cells, which include tissue-resident macrophages and dendritic cells. The result of this program is the generation of an antipathogen state and the production of a myriad of inflammatory cytokines, chemokines, biogenic amines, and eicosanoids [18] that induce a similar state in neighboring tissue cells.

Soluble inflammatory chemokines [19] and activated complement [20, 21] produced in response to pathogen sensing contribute to the attraction of additional innate immune cells such as neutrophils, NK cells, and monocytes to the site of infection [19, 22]. The recruited inflammatory cells encircle the damaged or infected cells and release more proinflammatory cytokines including tumor necrosis factor (TNF), IL-6, IL-12, and type I and II interferons (IFNs). Neutrophils also release DNA nets to trap free extracellular pathogens [23, 24], and NK cells attempt to lyse infected host cells through cytotoxic means [25, 26]. The innate inflammatory cytokine and cellular swarm attempts to contain the pathogen until highly specific, activated cells of the adaptive immune response are recruited to ultimately clear the infection [27]. If coordinated recruitment of innate and adaptive immunity fails to effectively control the pathogen, clinical disease will ensue. A major challenge for vaccine design is to mimic this inflammatory environment, which is needed to stimulate the generation of effective and robust immunity, without causing the immunopathology and tissue damage associated with clinical infection.

### 2.1. Pathogen Sensing

In order for the inflammatory events discussed above to occur, pathogens must be detected in compromised tissues. Many different subsets of classic dendritic cells, plasmacytoid dendritic cells, and macrophages [28, 29] are distributed throughout tissues in a network that facilitates immediate detection of both invading pathogens and the associated tissue damage [30, 31]. These sentinel innate cells sense pathogens and pathogen-associated tissue damage in a generic way through multiple distinct pathways [32]. They employ germ-line encoded pattern recognition receptors (PRRs) that recognize pathogen-associated molecular pattern (PAMPs) [32] and damage-associated molecular pathogens (DAMP) [33, 34] to detect changes in their environment [35, 36]. Recognition of pathogen-derived products such as lipopolysaccharide (LPS) by Toll-like receptors (TLR) 1, 2, and 4; flaggelin by TLR5; single stranded (ss) by TLRs 7 and 8; double-stranded (ds) RNA by TLR 3; and CpG DNA by TLR9 occurs either at the surface of the cell or within endoplasmic vesicles [37]. Host cell-derived danger signals or alarmins such as heat shock proteins, uric acid crystals, high-mobility group box 1, S100 proteins, serum amyloid A, and products of purine metabolism released from damaged or stressed cells are sensed by DAMP receptors such as RAGE, TLRs, and purinergic receptors [38, 39]. Recognition of PAMPs and DAMPs triggers the activation of signaling pathways that ultimately leads to the expression of the transcription factors NF-*κ*b, AP-1, and interferon regulatory factors (IRFs) [32, 40, 41]. These transcription factors control the expression of hundreds of immune defense response genes [18, 40, 42]. An attractive means to both tailor and enhance the generation of vaccine-induced immunity is through the use of adjuvants that selectively trigger PRR and DAMP receptors. Such adjuvants are currently being explored to improve the generation of adaptive immune responses to inactivated pathogen and protein-based vaccines [8–11].

Advancements in our knowledge of intracellular sensors of pathogens and host-derived stress products have revealed novel targets to modulate and improve vaccine efficacy [43, 44]. A number of intracellular sensors, including the nucleotide-binding-domain and leucine-rich-repeat- (NLR-) containing proteins [45, 46] and the AIM-like-receptor (ALR) proteins [47], trigger the inflammasome pathway. The activation of the inflammasome complex and the activation of caspase-1 enzymatic activity are best known for triggering maturation of the proforms of the cytokines IL-1 and IL-18 [48]. However, alternative outcomes such as phagosome maturation, autophagy, glycolysis, lipid metabolism, and oxidation of arachidonic acid to generate eicosanoid signaling molecules, as well as inflammatory pyroptotic cell death, can also be triggered [44]. IL-1 and IL-18, in their mature forms, are potent proinflammatory cytokines [49]. The importance of the inflammasome-sensing pathway and the production of IL-1 and IL-18 to effective pathogen defense is highlighted by the fact that many infectious organisms, such as viruses, that gain access to the cytosol encode proteins that attempt to evade detection by intracellular sensors [50].

Intracellular sensors interact with adaptor proteins such as apoptosis-associated speck-like protein containing a C-terminal caspase activation and recruitment domain (ASC) [51] to trigger the activation of the proteolytic functions of the caspase-1 enzyme. Triggers of caspase enzymatic activity are extensively reviewed elsewhere [44, 46, 52]. The discovery of noncanonical activation pathways involving caspases other than caspase-1 [44, 53], as well as the ability of the type I IFN, a pro- and anti-inflammatory cytokine [54], to both prime cells for cytosolic sensing [44] and inhibit NLR signaling [55] emphasizes the need to more fully understand the workings of the inflammasome complex before targeted modulators [56] can be employed to enhance the generation of vaccine-induced memory CD4 and CD8 T cell immune responses.

### 2.2. Inflammatory “Rheostats”

Under normal circumstances, inhibitory “innate immune rheostats” act to prevent unnecessary inflammation at barrier surfaces [57, 58]. Inflammatory responses in tissues are tempered in many ways via recognition of soluble as well as cell surface ligands. This includes the blockade of activating DAMP receptor signaling by tissue-derived factors such as surfactant proteins and mucins [59–62]. Inhibitory DAMP receptor and inhibitory cytosolic receptor triggering by host-derived ligands such as DNA is an additional example of how inflammatory responses are kept in check [63–65]. Ligation of cell surface receptors on monocytes and dendritic cells, such as CD200R by CD200 ligand, that trigger dampening signals [66] is yet an additional means by which inflammation is regulated. Lastly, suppression of NF-*κ*b activation by the release of mitochondrial H_2_O_2_ in lung APC [67] and the production of the anti-inflammatory cytokines IL-10 and TGF-beta by both regulatory T cells and tissue cells [68–70] mitigate inflammatory responses. The potent efficiency of IL-10 and TGB-beta in counterregulating inflammatory cytokine production as well as in inhibiting both costimulatory and major histocompatibility complex (MHC) molecule expression on antigen-presenting cells (APCs) likely explains why many pathogenic viruses encode homologues of inhibitory cytokines and inhibitory ligands to evade the innate immune response. Expression of IL-10 by Epstein-Barr virus (EBV) [71] and expression of the inhibitory ligand CD200 by cytomegalovirus (CVM) [72] are prime examples.

Safely overcoming these “rheostats” by targeted blockade of inhibitory molecules or by employing novel adjuvant formulations that facilitate the generation of protective local immunity through vaccination without causing damaging adverse effects is of paramount importance [73–75]. Indeed, the generation of overzealous inflammatory responses following pathogen or adjuvant stimulation has the potential to cause severe inflammatory disease [34, 76]. Individuals who possess well-characterized genetic polymorphisms in numerous inflammatory mediators and signaling molecules, such as those associated with chronic inflammatory diseases like psoriasis, ulcerative colitis, and Crohn's disease [77], are at increased risk for developing undesired inflammatory complications following vaccination. In addition to genetic predispositions, environmental factors, such as the microbiome, may also play a role in setting the inflammatory “rheostat” at mucosal surfaces [78–82]. Interestingly, individual-specific microbiota signatures have been shown to impact both disease susceptibility and severity via either innate or adaptive immune pathways [83].

The control of immune response gene expression by long noncoding (lnc) RNAs [84] is another recently described homeostatic mechanism that could be targeted to improve vaccine efficacy as well as for therapeutic control of inflammation. Depending on the cell type involved, binding of specific lnc RNAs to regulatory regions of immune response genes and the subsequent control of nucleosome positioning can either promote or actively repress inflammatory gene expression [85]. A number of long noncoding RNAs are dysregulated during viral infection [86, 87], and changes in their expression are being assessed for use as biomarkers of disease severity [88]. The control of inflammatory responses by noncoding RNAs could have an exciting future in tailoring host inflammatory responses.

In addition to the homeostatic mechanisms and negative feedback loops discussed above, which preserve vital functions of organs such as the lung and intestine, the timing of vaccination administration may also need to be taken into account. Patterns of expression of proteins such as IL-6, inflammatory monocyte chemokine ligand (CCL2), as well as Toll-like receptor (TLR) 9, which are regulated by circadian clock proteins [89], may explain why morning vaccine administration appears more effective than afternoon administration at inducing specific antibody in older adults [90]. Differences in the magnitude of inflammatory responses across seasons may also influence the efficacy of vaccination. A recent study found that the magnitude of the inflammatory cytokine response detected following stimulation of monocytes with different pathogen-derived products, including those from influenza A virus, differs in different seasons [91]. In the individuals studied, inflammatory cytokine responses were maximal during the summer months of June and July and weakest in winter months [91]. The authors speculate that the tendency to produce reduced levels of inflammatory cytokines such as IL-1, TNF, and IL-6 during the winter may impact an individual's susceptibility to pathogens such as influenza A during the flu season. How the efficiency of vaccination is affected by the seasonal changes warrants further investigation.

## 3. Inflammation and the Generation of Adaptive Immune Responses

To successfully generate protective immunity through vaccination, antigen-specific T cells must interact with activated APC displaying cognate antigen in the context of MHC. Such interactions result in the receipt of signal 1, the specific antigen, and signal 2, the costimulatory molecule-dependent signals, required for full T cell activation. Recognition of inflammatory cytokines by their corresponding cytokine receptors constitutes signal 3 that can amplify proliferation as well as effector functions in activated cells.

Foreign antigens introduced by vaccination must reach the secondary lymphoid organs in order for T cell activation to occur. Antigen is delivered to draining lymph nodes via the lymph in particulate form or within migrating tissue-resident antigen-presenting cells that have egressed from the inflammatory site [92]. Particulate antigens in the lymph are captured by specialized APCs that are strategically poised in the draining lymph nodes [93]. Larger-sized antigens are captured by lymph node dendritic cells that reside within the lymphatic sinus endothelium [93] or by subcapsular sinus macrophages [94, 95]. Smaller-sized antigens are transferred to lymph node follicle dendritic cells and B cells via a conduit system [96]. Once engulfed and processed, antigens are presented by antigen-presenting macrophages, dendritic cells, and/or B cells to naïve CD4 and CD8 T cells on MHC class II and class I molecules, respectively. Antigens that gain access to the circulation are delivered to the spleen via the blood and are detected in a similar fashion by the APCs that reside there.

Exposure to and engulfment of pathogen-derived products at the site of vaccination or infection activates APCs and triggers their production of inflammatory cytokines. Cohorts of APC, once activated, will begin to migrate towards lymphoid organ chemokines CCL19 and CCL21 in a CCR7-dependent fashion [97, 98]. Egress of tissue-resident APC from sites of infection is a rapid event, and migratory subsets can be detected in lymphoid organs within 14 to 24 hrs of antigen administration [99, 100]. Both tissue-resident dendritic cells and macrophages display migratory behavior upon activation [29, 101–103]. Interestingly, following infection with respiratory viruses such as influenza A virus, one APC subset, alveolar macrophages, becomes undetectable in the infected lung tissue until recruited monocytes are able reestablish the population [104]. It remains unclear, however, whether the inability to detect alveolar macrophages following influenza is the result of their complete egress out of the tissue, a switch in their surface marker phenotype in response to the inflammatory milieu, or because of their elimination by the viral infection [29, 102].

The lifespan of activated tissue-migratory APCs within draining lymph nodes, especially the dendritic cell subset, is relatively short [105], and optimal antigen presentation by such cells occurs within 24 hrs of tissue egress [99, 100]. In addition to functioning as APCs within the T cell zones [101, 106], migratory dendritic cells can also act as “cargo carriers” that deliver engulfed antigen to APC resident in lymphoid organs [107, 108]. Whether migratory or lymph node resident, APCs once activated express increased levels of MHC I and II molecules, as well as increased expression of costimulatory molecules, such as CD40, CD70, CD80, and CD86 [28]. Activated APCs also produce numerous proinflammatory cytokines including IL-12, IL-6, and type I IFN for the plasmacytoid dendritic cell subset [28]. The inflammatory mediators that these highly activated APCs produce and the surface costimulatory molecules that they express play a key role in shaping the ensuing adaptive immune response [109, 110]. Vaccine strategies that specifically target pathogen-derived antigens to APCs *in vivo* [111], that employ antigen-loaded dendritic cells themselves as the vaccine vehicles [112], or that additionally trigger specific PRR receptors to direct T cell polarization are actively being explored as means to amplify the generation of effective T cell responses [8–10]. Such strategies are of particular interest for vaccination regimes for the elderly and cancer patients where the generation of effective immunity is challenging because of their compromised or suppressed immune states [112, 113].

### 3.1. And Back Again: The Regulation of Early Innate Inflammatory Responses by Memory T Cells

Following an acute infection or vaccination, the activation and expansion of naïve pathogen-specific T cells and the generation of effector cells generally occur within 7 days. Under normal circumstances, the majority of expanded effector cells that migrate to sites of infection or antigen administration undergo contraction following subsequent pathogen or antigen clearance. A small cohort of the expanded effectors will, however, survive to memory [114]. These antigen-specific memory cells, which exist at a frequency higher than that found in the naïve state [115], mediate potent immunological protection upon secondary pathogen encounter.

Some antigen-specific memory T cells possess the ability to migrate throughout the body and are readily detected within tissues [116, 117]. This migration pattern is markedly different from that of naïve T cells, which only circulate through the blood and secondary lymphoid tissues [118, 119]. When compared to naïve T cells, memory T cells also have increased cytokine-producing potential [120, 121]. One subset of memory T cells, the tissue-resident memory T cell subset that does not circulate, is found exclusively within the tissues and may be strategically poised and specialized to perform sentinel functions [122–125]. Targeting the generation of tissue-resident memory T cells, especially for pathogens that infect mucosal tissues, is thus an attractive means to improve the efficacy of vaccines against pathogens that are not effectively controlled by traditional antibody-based approaches.

**Figure 1:**
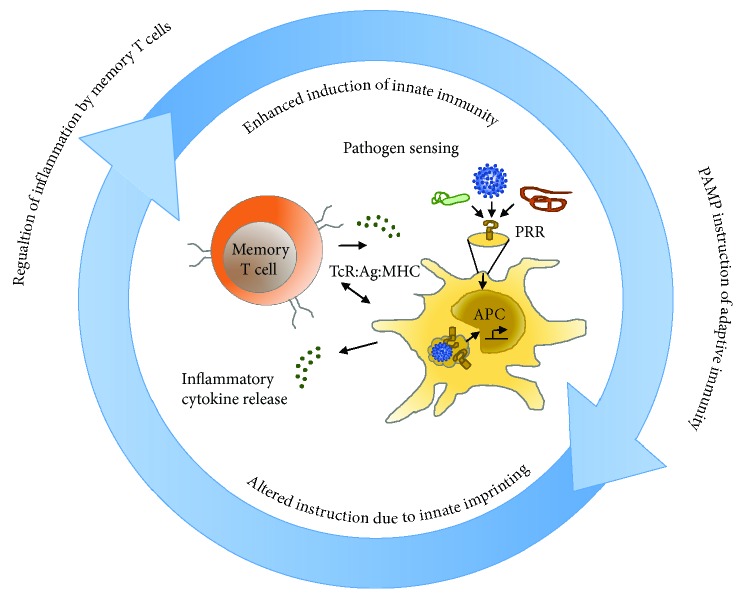
Memory T cells regulate inflammation at sites of infection. Traditional paradigms of innate instruction of adaptive immunity must now also appreciate that adaptive memory T cells regulate both the nature and shape of innate inflammatory responses. Memory CD4 T cell-mediated enhanced inflammatory responses are initiated independently of the classic PRR signaling and classic costimulatory molecule recognition and are better at containing virus than innate responses triggered in naïve hosts through PAMP-dependent mechanisms.

In addition to rapidly producing cytokines upon recognition of cognate antigen, memory T cells perform many other effector functions to protect the host against infection [126]. These functions are, for the most part, recalled independently of most costimulatory molecules [127]. This is one major way in which memory cells are distinct from naïve T cells that are dependent upon costimulatory signals for their full activation. For CD4 T cells, the best-known effector role is the provision of help for antigen-specific B [128] and cytotoxic CD8 T cell responses as reviewed elsewhere [126, 129, 130]. A novel effector role of memory T cells that is becoming more appreciated is the regulation of innate immune responses at sites of infection [126]. Of importance to this discussion, memory T cells mediate rapid production of effector cytokines akin to the responses elicited from innate immune cells upon cognate encounter with specific pathogen-derived antigen. Memory T cells thus have the potential to act as powerful antigen-specific sentinels that are able to initiate rapid inflammatory responses against pathogens [122, 131–133]. In fact, our studies in an influenza model showed that memory T cell-mediated inflammatory responses are induced faster, are bigger, and are better at containing virus than innate responses in naïve IAV-infected animals that are triggered through PRR-dependent mechanisms [133] (Figure 1).

Both memory CD4 [132, 133] and CD8 [131, 134, 135] T cells have the capacity to regulate and enhance the generation of early innate inflammatory responses within tissues upon cognate recognition of antigen. The antigen-specific regulation of inflammatory responses provides an additional means by which the immune response can generate alarm signals during infections with pathogens that possess means to evade detection by the innate immune-sensing mechanisms discussed earlier [136]. It also provides a means whereby experienced memory cells can modulate the effector functions of the ensuing adaptive response of expanded secondary effector T cells that arise from resting memory T cell precursors during recall [137].

For memory CD4 T cells, enhanced inflammatory responses are initiated in the lung following IAV infection independently of the classic PRR signaling molecules MYD88 and TRIF [133]. Memory CD4 T cell-regulated enhanced inflammatory responses can also be initiated in the absence of infection. Indeed, the intranasal administration of cognate peptide antigen in the absence of any adjuvants or the administration of endotoxin-free protein that contains the epitope for which the cells are specific leads to the generation of potent early innate inflammatory responses [133]. This suggests that even though CD4 T cells themselves can express PRRs and produce inflammatory cytokines following PAMP recognition [138, 139], such PRR triggering is not required for the mediation of memory CD4 T cell sentinel functions [133].

The ability of memory CD4 T cells to induce inflammatory responses upon pathogen detection is also independent of their production of the classic proinflammatory cytokines TNF and IFN-*γ* and does not require the receipt of CD80, CD86, and CD40 costimulatory molecule signals [133]. That memory cells do not depend on signal 2 to perform sentinel functions within the lung is in fitting with the observation that the activation and early recall of memory CD4 T cells *in vivo* are not affected by blockade of the CD28 costimulatory pathway [140]. The sentinel capacity of memory CD4 T cells thus appears to be very different from the sentinel functions of CD8 T cells, which are dependent upon TNF [141], IFN-*γ* [142–144], GM-CSF [145], and potentially also the receipt of costimulatory signals *in vivo* [146]. Similarities and inherent differences in the priming and function of memory CD4 and CD8 T cell responses are additional factors that must be considered in the design of innovative vaccination strategies that target the generation of protective antigen-specific T cells.

**Figure 2:**
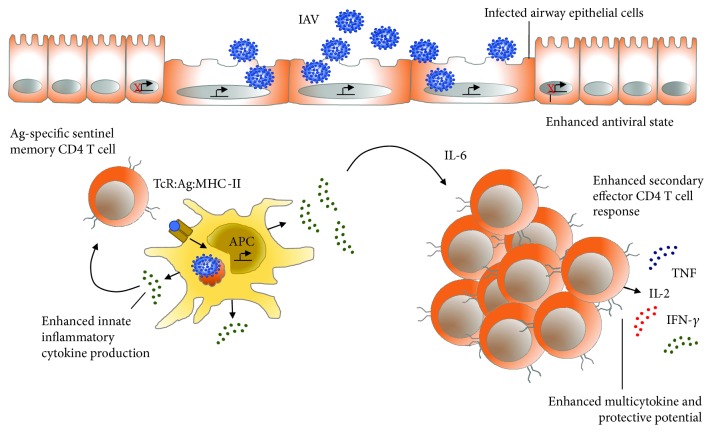
Memory T cells regulate the accumulation and functional potential of secondary effector T cells in the lung through antigen-specific upregulation of the inflammatory cytokine IL-6. Following secondary IAV infection, the earlier and more robust inflammatory response induced by memory CD4 T cells correlates with improved control of the virus in the lung. One innate inflammatory cytokine involved in this response, IL-6, plays a central role in maximizing the multicytokine-producing potential of secondary CD4^**+**^ T effector cells that accumulate in the lung at the peak of the recall response. Experienced memory cells thus modulate the effector functions of recently expanded secondary effector T cells that arise from resting memory T cell precursors during recall responses by inducing potent inflammatory signals.

Following secondary IAV infection, the earlier and more robust inflammatory response induced by memory CD4 T cells correlates with improved control of the virus in the lung [133]. Our recent findings show that one innate inflammatory cytokine involved in this response, IL-6, plays a central role in maximizing the multicytokine-producing potential of secondary CD4^**+**^ T effector cells that accumulate in the lung at the peak of the recall response [137] (Figure 2). In murine and human systems, multicytokine-producing potential, or the ability to coproduce TNF, IL-2, and IFN-*γ*, is associated with the ability of memory T cell responses to protect against numerous viral, bacterial, and parasitic pathogens [120]. Multicytokine potential, as well as the ability to mediate effector functions such as help and cytotoxicity, correlates with superior protective capacity when secondary effector cells (derived from memory precursors) are compared on a per cell basis to primary effectors derived from naïve T cells [121, 147, 148]. Innovative vaccines thus not only should target the induction of large numbers of memory T cells but also should strive to generate cells that possess optimal functional potential. Current research employing high-dimensional mass cytometry that simultaneously measures over 40 parameters, including cell surface markers and intracellular proteins, as well as RNA expression at single-cell resolution [149], will further advance our understanding of strong correlates of protection in specific models of infection. Such correlates will, in turn, help facilitate the development of optimal vaccination strategies.

## 4. Training of the Innate Immune System

Another significant advance in our understanding of innate immunity is the knowledge that cells of the innate immune system are altered or “trained” by past experiences [150]. For the majority of innate immune cells, such imprinting results in a generic and nonspecific heightened inflammatory response that increases host antimicrobial defenses upon secondary infection. Responses by NK cells may be an exception to this as they have been shown to display some elements of antigen-dependent memory [151–153]. It should be noted, however, that trained innate immune responses are functionally distinct from the highly specific recall responses characteristic of adaptive immune memory mediated by specialized subsets of CD4 and CD8 T cells and of antibody-producing B cells.

It has been long been appreciated that organs such as the lung remain in an altered state for an extended period of time following infection or insult [154, 155]. The heightened inflammatory state that exists following the resolution of pathogen infection lasts for days, weeks, or even months and can provide a degree of nonspecific protection to unrelated pathogens. Examples of heightened protective immunity induced by infection or vaccination are many and are discussed in detail elsewhere [150, 156, 157]. A prime example is the ability of BCG vaccination, in mice as well as in humans, to increase resistance against a number of different pathogens [157–160]. Priming of innate immune cells resulting in increased nonspecific pathogen protection can also be caused by viral pathogens [161, 162] and even exposure to pathogen-derived molecular patterns [163–166].

The protection afforded by “imprinted” innate immunity is associated with the presence of increased numbers of activated macrophages [150, 156], dendritic cells [167], and other innate immune cells within the tissues that are characterized as being in a heightened antimicrobial state [150, 156]. In animal models, this nonspecific protection is transferrable to naïve hosts by the adoptive transfer of “trained” macrophages, and, notably, the transfer of protection does not require the presence of T cells [165, 168]. Recent studies have shown that this “imprinted” state is maintained by long-term translational and epigenetic changes within the “trained” monocytes and macrophages [165, 169, 170]. Signals generated through recognition of the microbiota that ultimately lead to the production of the inflammatory cytokine GM-CSF, which also has colony-stimulating functions, is just one example of how heightened inflammatory “rheostats” can be established within mucosal tissues [171]. How conditioning of innate immune cells by the microbiota and infectious pathogens in human tissues influences the ability to generate protective immune responses following vaccination remains to be determined. However, some groups have begun to establish models using primary human monocytes to shed preclinical insight on the ability of pathogen-derived products to “imprint” human APC *in vitro* [172].

Pathogen-associated encounters may not be the only events capable of training innate immune cells. The engulfment of apoptotic host cells in the absence of infection has traditionally been considered an immunologically neutral event that fails to generate DAMP signals [33]. Recent observations, however, show that even this steady-state process can imprint macrophages for heightened inflammatory responses that mediate nonspecific resistance to microbial infection [173]. These and other findings in a murine model [174] suggest that most if not all tissue-resident macrophages become experienced during development by normal cellular turnover processes that educate them for future pathogen encounter.

The altered inflammatory state that exists following the resolution of infection can also have alternative and undesired outcomes. For example, conditioning of innate immune cells by prior infection can result in increased susceptibility to secondary infection [175]. Increased susceptibility to secondary bacterial infection occurs following many respiratory virus infections [176] and contributes markedly to the morbidity and mortality of disease [177]. Mechanisms underlying increased susceptibility to secondary infection are many and include deficiencies in bacterial scavenging receptors such as MARCO on macrophages [178], as well as the depletion of tissue-resident APC populations during primary infection [104]. Increased production of inflammatory dampening cytokines IL-10 and TGF-beta [179, 180] and attenuation of protective host defenses through diminished production of IL-1b [181], IL-27 [182], and antimicrobial peptides [181] can also contribute to increased susceptibility. Increased expression of inflammatory dampening receptors such as CD200R [66, 155] and differences in the chemotaxis, survival, phagocytic, and respiratory burst functions of neutrophils [183–185] may also lead to an inability of the innate immune system to contain and control secondary microbial threats following respiratory viral infection. In addition to regulating early inflammatory responses that facilitate pathogen control, vaccine-induced T cell immunity may also be able to prevent these deficiencies in innate immunity as experimental evidence suggests that susceptibility to secondary bacterial infections is mitigated in primed animals in models of IAV infection [186].

### 4.1. And Back Yet Again: Heterologous Infection, Memory T Cells, and Inflammation

While highly specific in nature, the adaptive immune response can also alter the outcome of infections with seemingly unrelated pathogens. This phenomenon, which has been termed heterologous immunity [187], is mediated by cross-reactive T cells with T cell receptors that have the potential to recognize more than one peptide-MHC complex. Heterologous immunity is long-lasting and much like “innate imprinting” it can be either beneficial or detrimental. For instance, in animal models of lymphocytic choriomeningitis virus (LCMV), cytomegalovirus (CMV), or IAV infection, prior virus-specific immunity has a beneficial impact on the outcome of subsequent vaccinia virus infection and results in improved viral clearance [188]. However, in the reverse scenario, prior IAV-specific immunity can increase the immunopathology of respiratory LCMV and murine CMV infection. Preexisting, heterologous immunity has been shown to alter protective T cell immunodominance hierarchies induced by primary infection. It is argued that the presence of cross-reactive T cells narrows the virus-specific T cell repertoire and drives the selection of viruses able to escape adaptive immunity. Conversely, the recall of cross-reactive memory T cells can also result in protective immune responses. Given the capacity of memory T cells to regulate inflammation [133, 135], beneficial heterologous immunity in the latter scenario likely also involves well-guided innate inflammatory responses that contribute to the initial control of pathogens. One can thus infer from these studies that the severity of disease is impacted not only by the past history of infections but also by the sequence of such infections. These observations have important implications for the design and timing of the delivery of vaccines [189].

## 5. Summary

Understanding of the impact of prior pathogen encounter on both innate and adaptive immunity is imperative for the design of innovative vaccination regimes. Exciting developments in the field of macrophage and monocyte biology are changing the way memory is typically perceived in innate immune cells. The “training” of innate immunity must be further investigated in order to effectively implement these insights into improved vaccines that are better able to promote durable memory states. In addition, traditional paradigms of innate instruction of adaptive immunity must now appreciate that memory T cells regulate both the nature and shape of innate inflammatory responses through antigen-specific means. Furthermore, memory-regulated inflammatory responses can impact the development and functional potential of secondary effector T cells. Every infection, commensal interaction, and immunogenic vaccine thus has the potential to change the host tissue microenvironment as well as the adaptive immune T cell repertoire. Such changes can impart lasting immunological consequences that are able to influence subsequent responses to infection both positively and negatively.
